# Redistribution of DAT/α-Synuclein Complexes Visualized by “In Situ” Proximity Ligation Assay in Transgenic Mice Modelling Early Parkinson's Disease

**DOI:** 10.1371/journal.pone.0027959

**Published:** 2011-12-07

**Authors:** Arianna Bellucci, Laura Navarria, Elisa Falarti, Michela Zaltieri, Federica Bono, Ginetta Collo, Maria Grazia, Cristina Missale, PierFranco Spano

**Affiliations:** 1 Division of Pharmacology, Department of Biomedical Sciences and Biotechnologies and National Institute of Neuroscience-Italy, School of Medicine, University of Brescia, Brescia, Italy; 2 Department of Clinical Neurosciences, Brain Repair Centre, University of Cambridge, Cambridge, United Kingdom; 3 Istituto Di Ricovero e Cura a Carattere Scientifico San Camillo, Venice, Italy; University of South Florida College of Medicine, United States of America

## Abstract

Alpha-synuclein, the major component of Lewy bodies, is thought to play a central role in the onset of synaptic dysfunctions in Parkinson's disease (PD). In particular, α-synuclein may affect dopaminergic neuron function as it interacts with a key protein modulating dopamine (DA) content at the synapse: the DA transporter (DAT). Indeed, recent evidence from our “in vitro” studies showed that α-synuclein aggregation decreases the expression and membrane trafficking of the DAT as the DAT is retained into α-synuclein-immunopositive inclusions. This notwithstanding, “in vivo” studies on PD animal models investigating whether DAT distribution is altered by the pathological overexpression and aggregation of α-synuclein are missing. By using the proximity ligation assay, a technique which allows the “in situ” visualization of protein-protein interactions, we studied the occurrence of alterations in the distribution of DAT/α-synuclein complexes in the SYN120 transgenic mouse model, showing insoluble α-synuclein aggregates into dopaminergic neurons of the nigrostriatal system, reduced striatal DA levels and an altered distribution of synaptic proteins in the striatum. We found that DAT/α-synuclein complexes were markedly redistributed in the striatum and substantia nigra of SYN120 mice. These alterations were accompanied by a significant increase of DAT striatal levels in transgenic animals when compared to wild type littermates. Our data indicate that, in the early pathogenesis of PD, α-synuclein acts as a fine modulator of the dopaminergic synapse by regulating the subcellular distribution of key proteins such as the DAT.

## Introduction

Parkinson's disease (PD) is characterized by a progressive loss of dopamine (DA) neurons of the nigrostriatal system and by the presence of Lewy bodies (LB), proteinaceous inclusions mainly composed by filamentous α-synuclein aggregates [1]–[3].

Alpha-synuclein is a natively unfolded protein which plays a central role in the control of dopaminergic neuronal functions [3]; [4] and which is thought to be critically implicated in PD pathophysiology. Indeed, besides the fact that α-synuclein is the main protein component of LB, genetic studies indicate that mutations and multiplications of the α-synuclein gene are responsible for the onset of familial forms of PD. Recent findings showed that decreased putaminal DA transporter (DAT) binding and DA deficits occur in patients bearing nigral α-synuclein burden [5] shading light upon the notion that, in the PD brain, α-synuclein deposition in the substantia nigra inversely correlates with striatal DAT functions. This is a relevant observation, as the DAT acts as a key modulator of dopaminergic signalling by mediating rapid clearance of DA from the synaptic cleft [6]; [7]. The DAT is localized both at synaptic and extra-synaptic sites in cell bodies and dendrites of dopaminergic neurons of the substantia nigra, as well as in dopaminergic terminals in the striatum [8]; [9]. At these locations, it mediates stimulated and quantal DA reuptake, thus controlling DA recycling at the synapse as well as the lifetime of DA spillover [6]; [10]; [11]. Therefore, to define whether and how α-synuclein may affect its function is crucial to unravel the molecular mechanisms underlying DA-related PD pathophysiology. Previous studies have shown that a direct protein-protein interaction between these two proteins occur [6]; [12]–[14]. In particular, the N-terminus of α-synuclein is known to bind the C-terminus of the DAT [12]; [13]. Remarkably, it has been found that this interaction is essential for the attenuation of DAT activity mediated by α-synuclein, a function which is thought to be relevant for the control of DA synaptic tone [13]; [15]; [16]. In particular, it seems that α-synuclein can negatively regulate DAT activity by tethering the transporter to the microtubular network, as agents which disrupt microtubular dynamics abolish the inhibitory effect of α-synuclein upon the DAT [17]. However, the evidence that α-synuclein is a negative regulator of the DAT has been recently brought into question by other findings showing that α-synuclein knock-out and null mice show reduced DAT expression and function and a significant increase in basal DA release [18]. Furthermore, whether the data by Sidhu and coworkers supported the cause of a neuroprotective role of α-synuclein through the control of DA influx, Lee and coauthors [12] found that the formation of α-synuclein-DAT complexes facilitates the membrane clustering of the DAT, thereby accelerating cellular DA uptake and DA-induced cellular apoptosis. Although results from the above cited investigations are quite contradictory, it has to be taken into account that their discrepancies may be due to different cellular and animal models used for the studies. This notwithstanding, since α-synuclein directly interacts with the DAT and this interaction is known to modulate DAT functionality, it emerges that pathological changes, increase and/or aggregation of α-synuclein may fatally affect nigro-striatal dopaminergic functions by modulating DAT subcellular localization. Prompted by this hypothesis, we recently aimed at investigating the mechanisms through which pathological α-synuclein changes may affect DAT function by using dopaminergic cellular systems. We found that α-synuclein aggregation decreases DAT membrane expression and that DAT and aggregated α-synuclein are colocalized within intracytoplasmic inclusions in dopaminergic cells [14]. Furthermore, we fond that agents which are known to stimulate DAT surface expression, such as DA D2/D3 receptor agonists or cocaine [19]–[22], are able to inhibit the formation of DAT/α-synuclein immunopositive inclusions and increase both DAT and α-synuclein membrane expression in dopaminergic cells [14]. These data indicate that α-synuclein and DAT share common trafficking mechanisms. [14]. Hence, the pathological aggregation of α-synuclein may alter DA neuron function by affecting DAT distribution, a concept which is further reinforced by other recent findings by our group. Indeed, we found the occurrence of an age-dependent redistribution of other DAT-regulating proteins: synaptic N-ethylmaleimide sensitive fusion attachment protein receptor proteins (SNAREs), which can mediate the rapid increase of DAT surface expression [23], in the striatum of a transgenic mouse line expressing truncated human α-synuclein (1-120) (SYN120 mice). These mice show an age dependent deposition of α-synuclein aggregates in nigrostriatal dopaminergic cells [24], a decrease in striatal DA release and reduced locomotion, occurring prior to a frank dopaminergic neurodegeneration [24]; [25]. For these characteristics, the SYN120 mice represent an ideal model for the study of the α-synuclein-related pathological changes in the early pathogenesis of PD. Interestingly, in that same research report, we found that the DAT was also markedly redistributed in the striatum of 12-month-old SYN120 mice when compared to wild type littermates, supporting the idea that the pathological deposition of α-synuclein can critically affect DAT subcellular localization.

We thus aimed at investigating whether the DAT is redistributed as a consequence of α-synuclein aggregation because of the direct protein-protein interaction between these two proteins. To this purpose, we studied the occurrence of changes in the distribution of the DAT/α-synuclein complexes in the striatum and substantia nigra of 12-month-old SYN120 transgenic mice [24]; [25] by using a technique which allows the visualization of heteromeric protein complexes: the proximity ligation assay (PLA). We found a marked redistribution of DAT/α-synuclein complexes in the transgenic mouse brain. These alterations were accompanied by a significant increase of DAT levels, suggesting that pathological α-synuclein accumulation is able to modulate both the levels and the localization of DAT protein.

## Methods

### 1 Cell cultures

Control and dopaminergic differentiated SH-SY5Y cells (SH-SY5Y+) [14] were used. Briefly, cells were grown to confluence in complete medium made up by Dulbecco's modified Eagle's medium supplemented with 10% of heat-inactivated new born calf serum, 100 µg/mL penicillin, 100 µg/mL streptomycin and 0.01 µM non-essential amino acids (Gibco). Cells were maintained at 37°C in a humidified atmosphere of 5% CO_2_ and 95% O_2_.

Differentiation was performed incubating the cells for 3 days in complete medium supplemented with 10 µM of retinoic acid (RA) (Sigma-Aldrich) and for the following 3 days in complete medium containing 80 nM of 12-O-tetradecanoyl-phorbol-13-acetate (TPA).

### 2 Glucose deprivation

Glucose deprivation (GD) was performed according to the protocol described by Bellucci et al., [14] with minor modifications. Briefly, SH-SY5Y+ cells were incubated for 15 min at 37°C in Dulbecco's modified Eagle's medium containing no glucose (Sigma-Aldrich) supplemented with 10% of new born calf serum and 0.01 µM nonessential amino acids (NEAA). Then this medium was removed and replaced with complete medium for 24 h. For the PLA, cells were fixed in 4% paraformadehyde at 24 h from GD.

### 3 Generation of SYN120 construct and cell transfection

The cDNA of human truncated SYN120 α-synuclein was produced as previously described [26]. For cell transfection SH-SY5Y cells were grown to 60–80% confluency and transfected with either 5 µg of the SYN120 or 5 µg of the empty vector using the Lipofectamine 2000 reagent (Invitrogen) acconding to the manufacturer instructions. The efficiency of transfection was assayed by Western blotting followed by the densitometric assay of bands and statistical analysis. A statistically significant increase of 68 % (P <0.05) of α-synuclein expression was found in the SYN120 transfected cells when compared to control SH-SY5Y+ cells (please see figure S1A,B for further information).

### 4 Animals

Mice transgenic for human α-syn(1–120) were produced on a C57BL/6S background (C57BL/6JOlaHsd, Harlan) which lacks mouse α-synuclein [24]; [27]. Transgenic homozygous SYN120, C57BL/6J and C57/BL6S mice of 12 months of age were used for fluorescence double labelling and PLA experiments or DAT semiquantitative determinations. Mice were bred in our animal house facility. The mice were housed in macrolon cages with ad lib food and water and maintained on a 12-h light/dark cycle at a room temperature of 23 °C. All experiments were carried out according to the Directive 2010/63/EU of the European Parliament and of the Council of 22 September 2010 on the protection of animals used for scientific purposes. All experimental and surgical procedures conformed to the National Research Guide for the Care and Use of Laboratory Animals and were approved by the Animal Research Committees of the University of Brescia (Protocol Permit number 04/10). All efforts were made to minimize animal suffering, to reduce the number of animals used and to utilize alternatives to “in vivo” techniques, if available. Expression of the transgene in SYN-120 mice was assayed by DNA extraction from tail samples and PCR analysis by using a couple of primers (5′-agggtgattcagaggcaggt-3′ and 5′-ctgctccctccactgtcttc-3′) recognizing a portion of the rat tyrosine hydroxylase promoter which is driving the expression of the SYN120 transgene (please see figure S1C for further information) as previously described [24].

### 5 Immunohistochemistry

For immunohistochemistry, SYN120 and control mice were anesthetized with chloral hydrate (400 mg/kg, i.p.) and were perfused transcardially with 4% ice-cold paraformaldehyde in 0.1 M phosphate buffer, pH 7.2. After 4 h of postfixation, brains were put in 18% sucrose for at least 24 h and then 30 µm coronal sections were cut with a cryostat. Single and double labelling immunohistochemistry was performed according to previously described methods [28]; [29]. The immunostaining intensity of DAT was analysed as optical density by using the NIH IMAGE J software (NIH, Bethesda, MD, USA). Ten striatal sections per animal were selected at 30 µm interval and were analysed and quantified blinded to the genotypes.

### 6 “In situ” Proximity Ligation Assay (PLA)

The “in situ” PLA studies on fixed cells and brain tissue were performed as follows (O-LINK Bioscience, Upsalla, Sweden). Briefly, fixed cells or brain slices were incubated with blocking solution for 30 min at 37°C and then with the primary antibodies recognizing DAT and α-synuclein at 1∶200 dilution overnight at 4°C. On the following day, samples were washed in low buffered Tris Buffered Saline with Tween 20 (TBS-T) that was prepared according to the O-LINK bioscience recipe. Then, the cells and brain slices were incubated with the PLA probe solution (containing the secondary antibodies conjugated with the DNA probes) for 120 min at 37°C. After the removal of the PLA probe solution samples were washed in TBS-T and incubated with the hybridization solution containing oligonucleotides that hybridize to the PLA probes for 15 min at 37°C. Then the samples were washed and subsequently incubated in the ligation solution (containing the DNA ligase which allows the ligation of the probes and oligonucleotides to form a round circle DNA strand) for 15 min at 37°C. Subsequently, samples were washed in TBS-T and incubated with the amplification solution, containing DNA polymerase for the rolling cyrcle amplification (RCA), at 37°C for 90 min. Finally, the samples were incubated with the detection stock solution (containing Texas Red labeled oligonucleotides that hybridize to the RCA product) for 60 min at 37°C, washed in SSC buffers (made up according to the manufacturer's recipe) and ethanol and then mounted and analyzed by means of a confocal microscope.

### 7 Western blot studies

For DAT and total α-synuclein extraction striatal brain tissue from C57BL/6J, C57BL/6S and SYN120 mice were lysed in TBS+ (50 mM Tris-HCl, pH 7.4, 175 mM NaCl, 5 mM EDTA, 0.1 mM PMSF, 1 mM *N*-ethylmaleimide, plus complete proteasome inhibitor mixture; Roche Diagnostics,Mannheim, Germany). Protein concentration in the samples were measured by using the Bradford assay (Pierce, Rockford, IL). Equal amounts of proteins (20–25 µg) were run on 4–12% Nu-PAGE Novex Bis-Tris gels (Invitrogen, Milan, Italy). Densitometric analysis of bands was performed by means of Gel Pro Analyzer version 6.0 (MediaCybernetics, Bethesda, MD, U.S.A.). All bands were normalized to Glyceraldehyde-3-phosphate dehydrogenase (GAPDH) levels as a control of equal loading of samples.

### 8 Antibodies

Alpha-synuclein was visualized by using syn-1 (BD-Bioscience, Milan, Italy) recognizing residues 121–125 of the human form and residues 91–99 of the human and mouse form [30] of α-synuclein. Anti-DAT, anti-synapsin Ia/b (Calbiochem, San Diego, CA, USA) and CREB-2 (Santa Cruz Biotechnology, Santa Cruz, CA, USA) polyclonal antibodies were used to visualize the respective substrates.

### 9 Microscopy

Fixed cells and mouse brain sections were observed by means of an inverted light/epifluorescence microscope (Olympus IX50; Olympus, Milan, Italy) or by means of a Zeiss confocal laser microscope (Carl Zeiss S.p.A., Milan, Italy), with the laser set on λ = 405–488–543 nm and the height of the sections scanning = 1 µm. Images (512×512 pixels) were then reconstructed using LSM Image Examiner (Carl Zeiss S.p.A) and Adobe Photoshop 7.0 (Adobe system, Mountain View, CA, USA) software.

### 10 [^3^H]-DA uptake assay

[3H]-DA uptake assay was performed on SH-SY5Y+ cells and SH-SY5Y+ cells subjected to GD. Briefly, the medium was removed and cells were washed twice with 37°C Krebs-Ringer-Solution (KRS) (16 mM NaH2PO4, 119 mM NaCl, 4.7 mM KCl, 1.8 mM CaCl2, 1,2 mM MgSO4, 1,3 mM EDTA, 5,6 mM Glucose, 1 mM L-Ascorbic Acid, pH 7,4). SH-SY5Y+ cells and SH-SY5Y+ cells subjected to GD were than incubated in triplicate at 37°C in KRS in absence or presence of 10 µM cocaine for 5 min. Then, [3H]-DA was added to the medium and incubated for 15 min at 37°C. The medium was then remuved and the cells were washed three times with ice-cold KRB. Finally, cells were collected in NaOH 1N and [3H]-DA was quantified by using scintillation cocktail in β-counter. Specific [3H]-DA uptake was calculated as a ratio between % [3H]-DA levels in cell lysates measured in basal conditions and in the presence of 10 µM cocaine (please see figure S5 for further information).

### 11 [3H]-DA release assay

[3H]-DA release was assessed in SH-SY5Y+ cells and SH-SY5Y+ cells subjected to GD. Briefly, the medium was removed and cells were washed three times with 37°C Basal-KRS (119 mM NaCl, 2,5 mM KCl, 2,5 mM CaCl2, 1,3 mM MgSO4, 1 mM NaH2PO4, 26,2 mM NaHCO3, 10 mM Glucose, 1 mM L-Ascorbic Acid, pH 7,4). Cells were then pre-loaded with [3H]-DA for 15 min at 37°C. Later, cells were quikly washed with B-KRS at 37°C and immediately treated with B-KRS or K+-KRS (69 mM NaCl, 5 M KCl, 2,5 mM CaCl2, 1,3 mM MgSO4, 1 mM NaH2PO4, 26,2 mM NaHCO3, 10 mM Glucose, 1 mM L-Ascorbic Acid, pH 7,4) or B-KRS supplemented with 10 µM tetrodotoxin (TTX). Every 10 min samples were collected for a total session of 30 minutes. Then, whole medium were picked up, washed twice and cells were collected in 1N NaOH. [3H]-DA was quantified by using scintillation cocktail in β-counter. The percentage of [3H]-DA released in the medium was determined as a % ratio between the D.P.M assayed in the media end the total D.P.M. assayed in the cells (released fractions plus cell lysate fractions, please see File S1 for further information).

### 12 Statistical analysis

Differences in striatal DAT levels as well as DAT O.D. between 12 month-old C57BL/6J, SYN120 and C57BL/6S mice were assayed by one-way ANOVA followed by Tukey's multiple comparison test. n = 5,6 for each group. Differences between [^3^H]-DA uptake (% basal/cocaine ratio) were analyzed by using the Student's t-test (n = 8,9 for each group). Differences between % [^3^H]-DA release from SH-SY5Y+ cells and glucose deprived SH-SY5Y+ cells in basal conditions and after K^+^ or TTX treatment were analyzed by two-way ANOVA followed by Bonferroni's post-comparison test (n = 7–9 for each group).

## Results

### 1 Detection of DAT and α-synuclein interaction by PLA “in vitro”

In order to assess whether DAT and α-synuclein complexes are detectable by the *in situ* PLA we used an “in vitro” cell system which has been extensively characterized by our group: dopaminergic differentiated SH-SY5Y (SH-SY5Y+) cells subjected to glucose deprivation, where we previously demonstrated the occurrence of DAT interaction with both full length and truncated α-synuclein [14]. SH-SY5Y+ cells express increased levels of endogenous DAT and α-synuclein afterwards dopaminergic differentiation with all-trans-retinoic acid (RA) and 12-O-tetradecanoyl-phorbol-13-acetate (TPA) [14]; [31]. We found that in these cells, glucose deprivation (GD) stimulated α-synuclein aggregation and that this event decreased DAT membrane levels, as the DAT and α-synuclein are co-localized in intracytoplasmic inclusions following the GD insult [14]. Therefore, this cellular system possesses ideal characteristics to evaluate whether DAT/α-synuclein interaction is appreciable “in situ” by the PLA, and whether alterations in the localization of DAT/α-synuclein complexes can be visualized by using this method.

The DuolinkTM *in situ* PLA is capable of detecting protein-protein interactions in tissue and cell samples prepared for microscopy [32]. The method is based on the use of two primary antibodies, raised in different species, that are recognizing the two proteins of the target interaction. A pair of oligonucleotide labeled secondary antibodies (PLA probes) is applied on the sample and a signal is generated only when between the two PLA probes there is a tight nearness. Indeed, the oligonucleotidic probes-labeled secondary antibodies are hybridized with two oligonucleotides which are then ligated into a closed circle when the PLA probes are in close proximity. This round circle oligonicleotide sequence is then amplified generating a concatemeric product extending from the oligonucleotide arm of the PLA probe. Subsequently, this product is hybridized with a mixture of fluorescent labeled probes thus allowing the amplification of the signal. As a consequence a single protein-protein interaction can be visualized *in situ* as a red dot by fluorescence microscopy. Hence, this technology can detect any antigen with proximate epitopes at the single molecule level [32]; [33].

**Figure 1:**
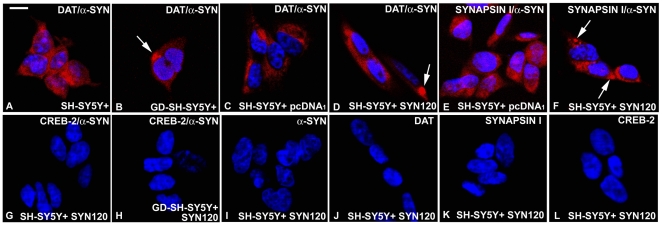
Detection of DAT and α-synuclein interaction by PLA “in vitro.” A: DAT and α-synuclein PLA in control SH-SY5Y+ cells. Please note a diffuse PLA-positive red signal. B: DAT and α-synuclein PLA in glucose deprived SH-SY5Y+ cells. Please note the presence of a PLA-positive inclusion (indicated by the arrow) within one of these cells. C: DAT and α-synuclein PLA in control SH-SY5Y+ cells. The PLA signal is weak and diffuse in these cells. D: DAT and α-synuclein PLA in SYN120-transfected SH-SY5Y+ cells. Please note the presence of an inclusion (indicated by the arrow) within one of these cells. E: synapsin I and α-synuclein PLA on pcDNA_1_-transfected SH-SY5Y cells. Please note that the PLA signal appears to be weak and diffused. F: synapsin I and α-synuclein PLA in SYN120-transfected SH-SY5Y+ cells. Some positive dots (indicated by the arrows) were visible within these cells. G: CREB-2 and α-synuclein PLA in control SH-SY5Y+ cells. No PLA signal was detected in these cells, which is indicative of the absence of CREB-2 and α-synuclein interaction. H: CREB-2 and α-synuclein PLA in glucose deprived-SYN120-transfected SH-SY5Y+ cells. No PLA signal is visible. I-L: Single labeled cells were used as negative controls for PLA experiments. I: α-synuclein antibody; J: DAT antibody; K: synapsin I antibody; L: CREB-2 antibody. Scale bar: 15 µm for A–L.

We thus investigated DAT/α-synuclein interactions in the SH-SY5Y+ dopaminergic cell model. We found that SH-SY5Y+ cells showed a diffuse PLA-positive signal, indicative of DAT/α-synuclein interaction. In particular, the DAT/α-synuclein PLA-positive signal was mainly localized at the periphery of the cell (fig. 1A) in line with our previous findings showing that these proteins were mainly localized on the cell membrane in the control SH-SY5Y+ cells [14]. Furthermore, in agreement with the previous observations showing that GD was able to induce the formation of DAT/α-synuclein-positive inclusions in SH-SY5Y+ cells [14], we found the presence of an intense PLA-positive signal into intracytoplasmic dot-like inclusions (fig. 1B) in the glucose deprived SH-SY5Y+ cells, thus confirming that the DAT is retained into intracytoplasmic inclusions following α-synuclein aggregation as these two proteins directly interact.

Then, we aimed at evaluating whether the overexpression of human c-terminally truncated (1–120) α-synuclein SYN120, which is expressed by the SYN120 transgenic mice, may affect the subcellular localization of DAT/α-synuclein complexes. To date, about 15% of α-synuclein in LB is truncated [34]–[37]. The pathological c-terminal cleavage of α-synuclein confers to the protein an high aggregation propensity [38]; [39]. This form of α-synuclein derives from the caspase like 20 S proteasomal degradation of unstructures full length intracellular α-synuclein [36]; [38] and it is believed to be implicated in the initiation and progression of the aggregation process of full length α-synuclein as it can seed the aggregation of the wild type form of the protein [36]–[38]; [40]; [41]. We thus investigated whether transfection of the human SYN120 α-synuclein construct, which coincides with the formation of intracellular aggregates “in vitro” [26], may affect the subcellular localization of DAT/α-synuclein complexes visualized by the *in situ* PLA. We found the presence of dot-like DAT/α-synuclein PLA-positive intracytoplasmic inclusions in the SYN120-transfected SH-SY5Y+ cells (fig. 1D). Similarly to the control SH-SY5Y+ cells, the cells which were transfected with the empty pcDNA_1_ vector showed a diffuse PLA-positive signal in proximity of the cell membrane, but no inclusions. Thus, our findings indicate that the overexpression of the (1–120) c-terminally truncated form of α-synuclein induces the formation of intracellular inclusions containing the DAT, and is consequently able to alter the subcellular distribution of the DAT.

To confirm that DAT levels on the plasma membrane were decrease in the SH-SY5+ cell subjected to glucose deprivation and in the SYN120-trensfected cells we performed immunoprecipitation experiments (please see File S11 for further information) using plasma membrane extract from control, glucose deprived- and SYN120-transfected SH-SY5Y+ cells. Our results showed that DAT and α-synuclein levels on the plasma membrane in the SH-SY5Y+ cells subjected to GD and transfected with the SYN120 construct were lower when compared to control cells (figure S2A).

In order to confirm the specificity of detection of DAT and α-synuclein interaction by the PLA, we performed a panel of control experiments to investigate whether synapsin I, a synaptic protein which is known to be part of DAT [42] and α-synuclein [43] proteome and which can also directly interact with α-synuclein in SH-SY5Y+ cells (figure S2B), may also be found in close proximity to α-synuclein within intracellular aggregates when this cellular system is transfected with the SYN120 construct. In particular, we aimed at evaluating whether α-synuclein/synapsin I interaction can be visualized *in situ* by the PLA and whether the distribution of this complex is also appreciable by the PLA. We were able to detect a dot-like positive signal, indicative of the close proximity of truncated α-synuclein and synapsin I within intracellular inclusions, in the SYN120-transfected SH-SY5Y+ cells (fig. 1F). In the control SH-SY5Y+ cells transfected with the empty pcDNA_1_ vector only a slightly detectable and diffuse PLA signal was present (fig. 1E). These observations confirm that the PLA signal is capable of detecting specific protein-protein interactions and indicate that α-synuclein aggregation may affect the correct subcellular distribution of other protein member of the DAT/α-synuclein proteome.

To further confirm that our PLA results were indicative of protein proximity we performed experiments to verify the absence of protein-protein interactions between α-synuclein and CREB-2, a transcription factor which didn't co-immunoprecipitate with α-synuclein in SYN120-transfected glucose deprived SH-SY5Y+ cells (please see figure S2C for further information). No interaction between α-synuclein and CREB-2 was detected in the glucose deprived SYN120-transfected SH-SY5Y+ cells (fig. 1H) where the GD insult is able to induce CREB-2 production [26] as in the control SYN-120-transfected SH-SY5Y+ cells (fig. 1G) which only express low levels of CREB-2 [26].

Finally, we performed control experiments by incubating SYN120-transfected SH-SY5Y+ cells with only one of the antibodies that we used for the previously describe PLA experiments. No PLA-positive signal was detected in the samples which were incubated with the sole anti-syn-1 (fig. 1I), anti-DAT (fig. 1J), anti synapsin-I (fig. 1K) or anti-CREB-2 (fig. 1L) antibodies.

Overall, these findings confirm that α-synuclein aggregation affects the proper localization of the DAT, and that the PLA is a useful technique to specifically visualize DAT/α-synuclein complexes and their subcellular distribution. In particular, we found that the DAT is retained within α-synuclein intracytoplasmic inclusions following the pathological aggregation of this latter, and these inclusions are visible by using the PLA.

### 2 DAT and α-synuclein labeling in the striatum and substantia nigra of wt and tg mice

The above described results prompted us to investigate whether the pathological aggregation of α-synuclein may alter the correct distribution of the DAT “in vivo”. We thus performed *in situ* PLA studies in a transgenic mouse line displaying α-synuclein aggregation into dopaminergic neurons of the substantia nigra: the SYN120 mice. This mouse model [24]; [25] express human c-terminally truncated (1–120) α-synuclein under the guidance of the rat TH promoter on an endogenous α-synuclein null C57BL/6S background [27]. At 12-months of age, the SYN120 transgenic mice show an age dependent decrease in striatal DA release and reduced locomotion, similar to PD [24]; [25], which are indicative of nigro-strital dopaminergic dysfunctions. To date, in a recent report, we already reported a marked redistribution of DAT protein in the striatum of this mice [25]. We thus considered that 12 month-old SYN120 mice represent an ideal model to investigate the redistribution of DAT/α-synuclein complexes. Twelve month-old C57BL/6J mice, expressing endogenous α-synuclein, and C57BL/6S littermates [27] which are null for endogenous α-synuclein, were used as controls.

**Figure 2:**
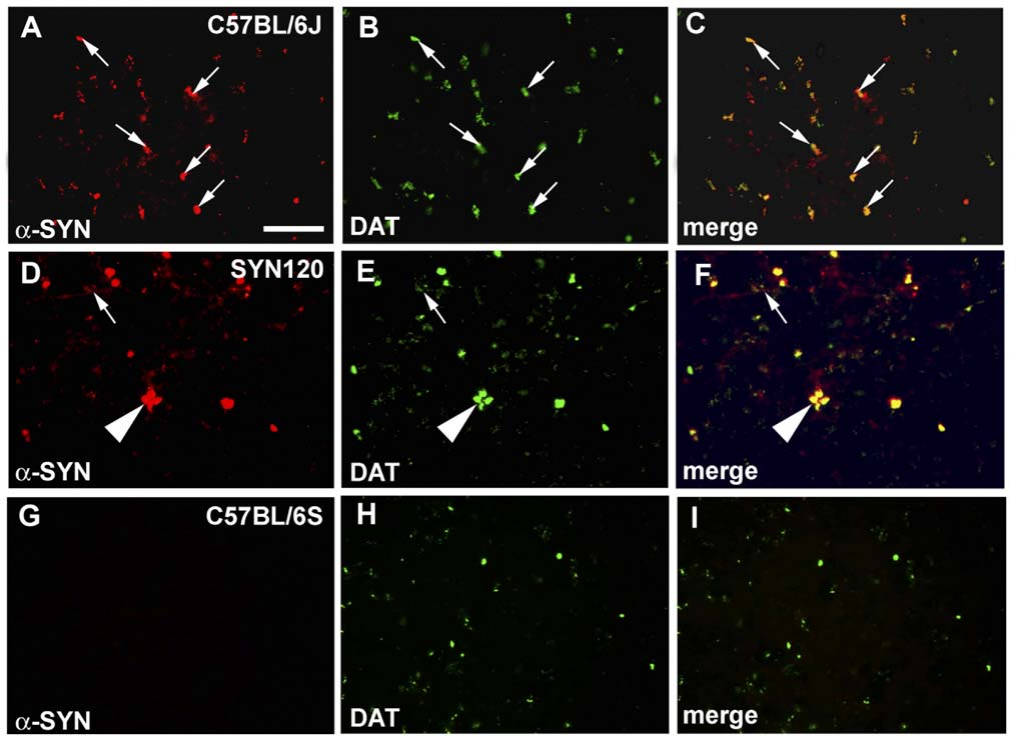
DAT and α-synuclein double staining in the striatum. DAT (green signal) and α-synuclein (red signal) double staining in the striatum of C57BL/6J (panel A–C), SYN120 (panel D–F) and C57BL/6S (panel G–I) mice. Panels C, F, I show the merges of DAT and α-synuclein labeling. Please note the marked redistribution of both DAT and α-synuclein immunolabeling in the SYN120 mice. In the C57BL/6S mice which didn't express α-synuclein, DAT distribution was similar to that observed in the C57BL/6J mice. Scale bar: 60 µm for A–I.

Firstly, we investigated both DAT and α-synuclein localization in the striatum and substantia nigra of 12 month old SYN120, C57BL/6J and C57BL/6S mice by fluorescence double labeling immunohistochemistry. We found that DAT and α-synuclein labeling almost completely co-localized in striatal dopaminergic terminals (arrows in fig. 2A–C) of the C57BL/6J mice, although a small portion of DAT staining, that didn't co-localize with α-synuclein, was diffusely distributed throughout the striatum fig. 2B–C. As in the C57BL/6J mouse brain, in the striatum of the SYN120 mice DAT and α-synuclein labeling partially colocalized. However, in these mice the distribution of DAT/α-synuclein labeling appeared to be different. Indeed, DAT and α-synuclein clustered in big dot-like inclusions (fig. 2D–F arrowhead) and neurite-like structures (fig. 2D–F, arrow) that were reminiscent of the α-synuclein-positive dots and of the Lewy neurites (LN) which have been described in the PD brain [44]–[46]. In the striatum of the C57BL/6S α-synuclein null mice we didn't observe α-synuclein staining and the DAT labeling displayed a distribution that was similar to that observed in the striatum of C57BL/6J mice (Fig. 2G–I).

**Figure 3:**
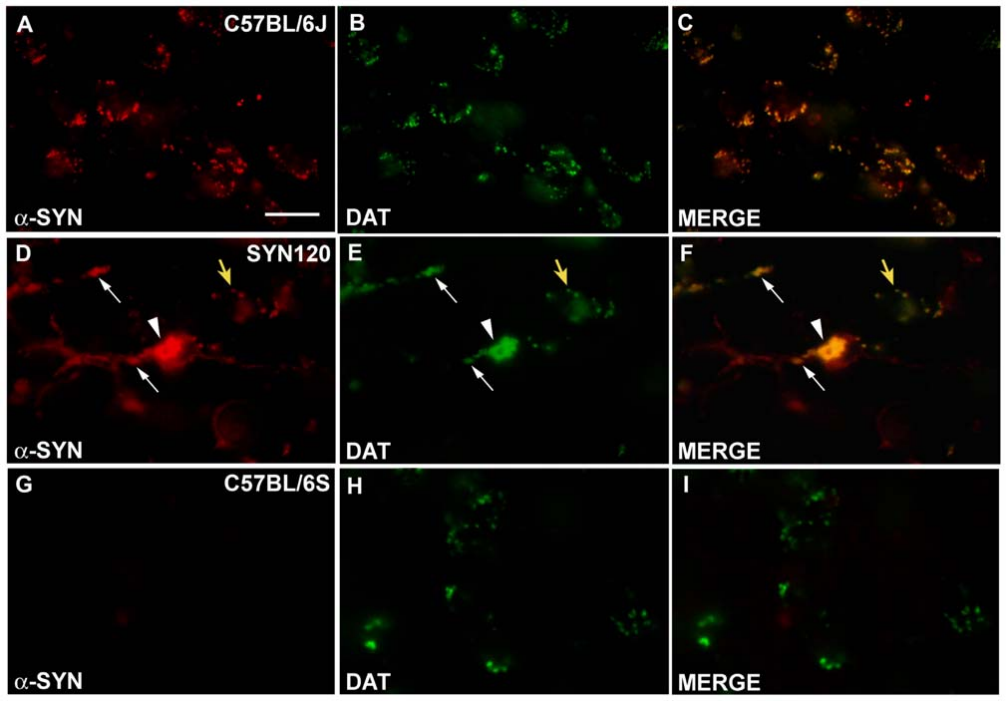
DAT and α-synuclein double staining in the substantia nigra. DAT (green signal) and α-synuclein (red signal) double staining in the substantia nigra of C57BL/6J (panel A–C), SYN120 (panel D–F) and C57BL/6S (panel G–I) mice. Panels C, F and I show the merges between DAT and α-synuclein immunolabelings. Please note that DAT and α-synuclein co-localized in dot-like clusters in the C57BL/6J mice. In the SYN120 mice DAT labeling was concentrated in big intracellular inclusions and within neuronal processes (arrows) and cell bodies (arrowhead) together with truncated α-synuclein, although some cells showed the same clustered co-localization of DAT and α-synuclein that we observed in the C57BL/6J mice (yellow arrow). In the C57BL/6S mice DAT distribution in the substantia was similar to that observed in the C57BL/6S mice. Scale bar: 40 µm for A–I.

In the substantia nigra of C57BL/6J mice, DAT and α-synuclein displayed a punctuate staining which appeared to be distributed on the cell membrane of dopaminergic neurons (Fig. 3A–C) in agreement with previous observations showing that DAT staining in this area of the mouse brain is mainly present on the cell membrane of dopaminergic neurons [9]; [47]. This observation was confirmed by immunofluorescent experiments which showed that DAT localization was similar to that of the membrane associated protein APP (please see figure S3 for further information). We also found that, as observed in the striatal sections, DAT and α-synuclein labellings almost completely colocalized (fig. 3C). In the substantia nigra of the SYN120 mice we observed that several neurons showed the same pattern of distribution of DAT and α-synuclein staining that we observed in the C57BL/6J mice, as they co-localized in dot-like structures on the plasma membrane (indicated by the yellow arrow in fig. 3D–F). However, in the neurons which contained α-synuclein inclusions, DAT staining was absent on the plasma membrane but it was intense in correspondence of the α-synuclein-positive aggregates in the cytoplasm (arrowhead) and in the processes (white arrows) as showed by representative photomicrographs in figure 3D–F. This distribution is reminiscent of the intracellular localization of DAT/α-synuclein inclusions that we previously described in glucose deprived dopaminergic cells [14]. Remarkably, in the SYN120 transgenic mice, α-synuclein labeling, although very intense in correspondence of intracellular inclusions, was still abundant on the plasma membrane, in agreement with previous findings indicating that truncated α-synuclein has an high propensity to interact with plasma membranes as it easily adopts an α-helical structure [37]. Finally, in the substantia nigra of the C57BL/6S mice α-synuclein labeling was absent (fig. 3G–I) and DAT staining was similar to that observed in the C57BL/6J wt mice as it displayed a punctuate distribution (Fig. 3H–I). These findings indicate that the subcellular localization of the DAT mostly reflected that of α-synuclein although a portion of these protein didn't co-localize, thus indicating that an elevated quote of these two proteins directly interact.

### 3 The localization of DAT and α-synuclein PLA signal was altered in the striatum and substantia nigra of SYN120 transgenic mice

To confirm the occurrence of DAT/α-synuclein interaction in the SYN120 and C57BL/6J mice we performed co-immunoprecipitation experiments (please see File S1 for further information) by using striatal protein extracts. C57BL/6S α-synuclein null mice were used as negative controls. We found that α-synuclein was present in the DAT-immunoprecipitates of the SYN120 and C57BL/6J mice, while in the striatum of C57BL/6S mice no α-synuclein was detected (figure S4).

To visualize alterations in the distribution of DAT and α-synuclein complexes in the striatum and substantia nigra of the 12 month-old C57BL/6J and SYN120 mice we used the “in situ” PLA. Again, the C57BL/6S mouse line lacking α-synuclein was used as negative control.

**Figure 4:**
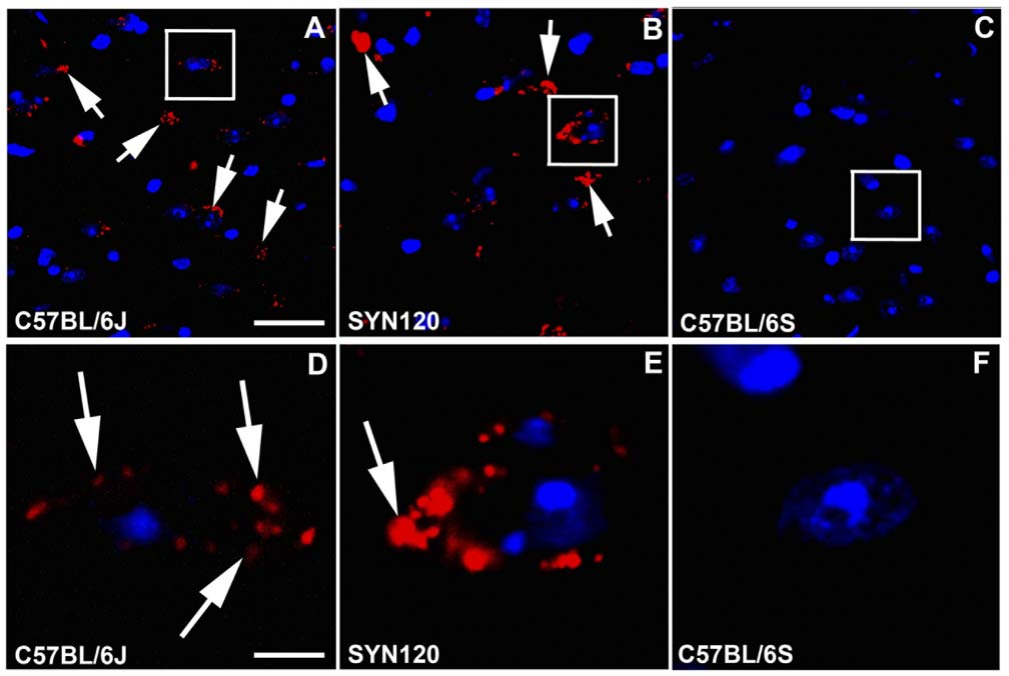
DAT and α-synuclein PLA in the striatum. DAT and α-synuclein PLA in the striatum of C57BL/6J (panel A) SYN120 (panel B) and C57BL/6S (panel C) mice. Panels D, E and F show higher magnifications of the squares in panels A, B and C, respectively. Please note that in the SYN120 mice the PLA signal (indicated by the arrows) displayed a more condensed distribution with respect to the C57BL/6J mice. In the C57BL/6S mice, which were lacking α-synuclein, no PLA signal was detected. Scale bars: A = 60 µm for A–C; D = 10 µm for D–F.

We found the occurrence of DAT and α-synuclein interactions in little dot-like structures (fig. 4A, D) in the C57BL/6J mice. In the striatum of the SYN120 mice DAT and α-synuclein PLA signal displayed an altered distribution as it appeared to be condensed in bigger aggregates (fig. 4B, E). In the α-synuclein null mice we didn't observe the PLA signal in the striatum, confirming the absence of the interaction between the proteins (fig. 4C,F).

**Figure 5:**
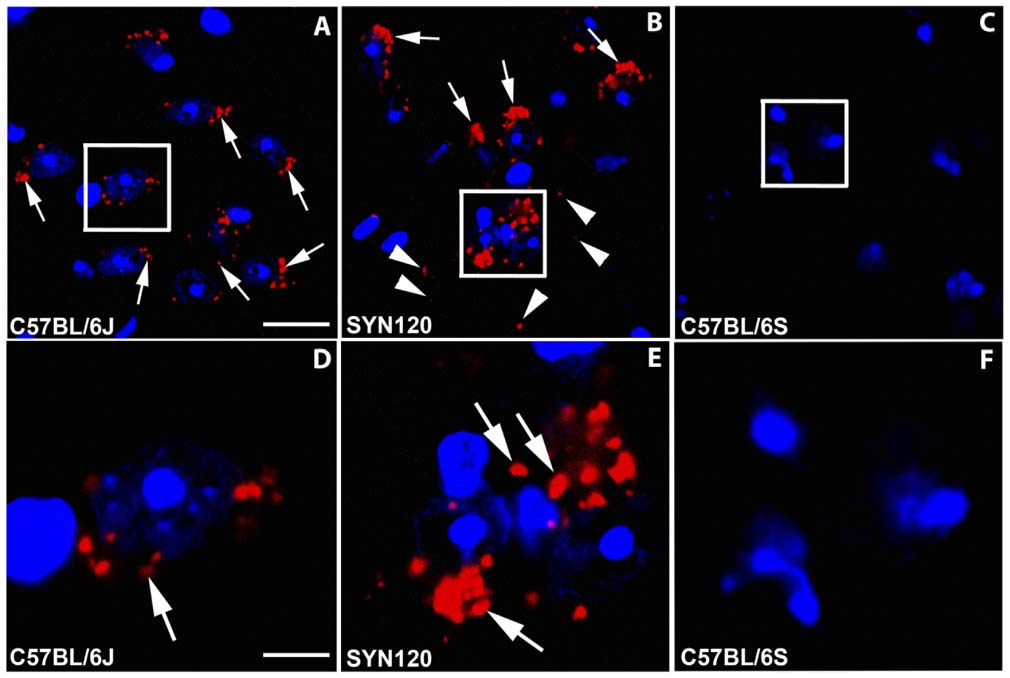
DAT and α-synuclein PLA signal in the substantia nigra. DAT and α-synuclein PLA in the substantia nigra of C57BL/6J (panel A, D) SYN120 (panel B, E) and C57BL/6S (panel C, F) mice. Panels D, E and F show higher magnifications of the squares in panels A, B and C, respectively. In the C57BL/6J mice PLA-positive blobs were present while in SYN120 mice the PLA signal was condensed in big dots. In the C57BL/6S mice no PLA signal was detected. Scale bars: A = 60 µm for A–C; D = 20 µm for D–F.

In the substantia nigra of the C57BL/6J wt mice an evident PLA-positive signal indicative of DAT and α-synuclein interaction was present. In particular, the PLA signal displayed a punctuate staining which followed the same localization of DAT and α-synuclein that we previously visualized by fluorescence immunoistochemistry (fig. 5A, D).

In the substantia nigra of the SYN120 mice the PLA signal clustered in big inclusions (fig. 5B, E). Furthermore, as observed by fluorescent staining, a small proportion of punctuate signal (indicated by the arrowheads in fig. 5B), reminiscent of the dot-like morphology of Lewy neurites which have been observed in the PD brain [44]–[46]; [48] was also detectable in the tissue.

In the substantia nigra of the α-synuclein null mice no PLA positive signal was detected, indicating the absence of protein-protein interaction between DAT and α-synuclein (fig. 5C,F).

### 4 DAT levels in the striatum of transgenic and control mice

**Figure 6:**
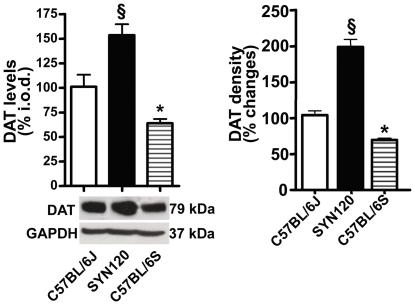
DAT levels in the striatum. A: Semiquantitative analysis of DAT levels in the striatum of C57BL/6J (white bars), SYN120 (black bars) and C57BL/6S (striped bars) mice. Please note the statistically significant increase of DAT levels in the SYN120 transgenic mice when compared to C57BL/6J and C57BL/6S wild type mice. B: Quantitative analysis of DAT immunoreactivity (% optical density) in the striatum of SYN120, C57BL/6J and C57BL/6S mice.

DAT levels in the striatum were assayed by semiquantitative analysis of DAT immunopositive bands from WB experiments (fig. 6). We found that in the 12 month-old SYN120 mice DAT levels were significantly increased respect to the C57BL/6J mice (§ +59%; P <0.001) and to the α-synuclein null mice (* +103%; P <0.001). Remarkably, DAT levels in the C57BL/6S mice were significantly lower (−44%, P <0.01) when compared to the C57BL/6J mice.

To confirm these findings we also quantified DAT immunoreactivity by using an image analysis software. We found a significant increase of % DAT density in the striatum of SYN120 transgenic mice when compared to the C57BL/6J (+95%, P <0.001) or to the C57BL/6S (+129%, <0.001) mice. Conversely, DAT density was significantly decreased in the striatum of C57BL/6S mice (−34%, P <0.05) with respect to C57BL/6J mice.

### 5 Functional studies

**Figure 7:**
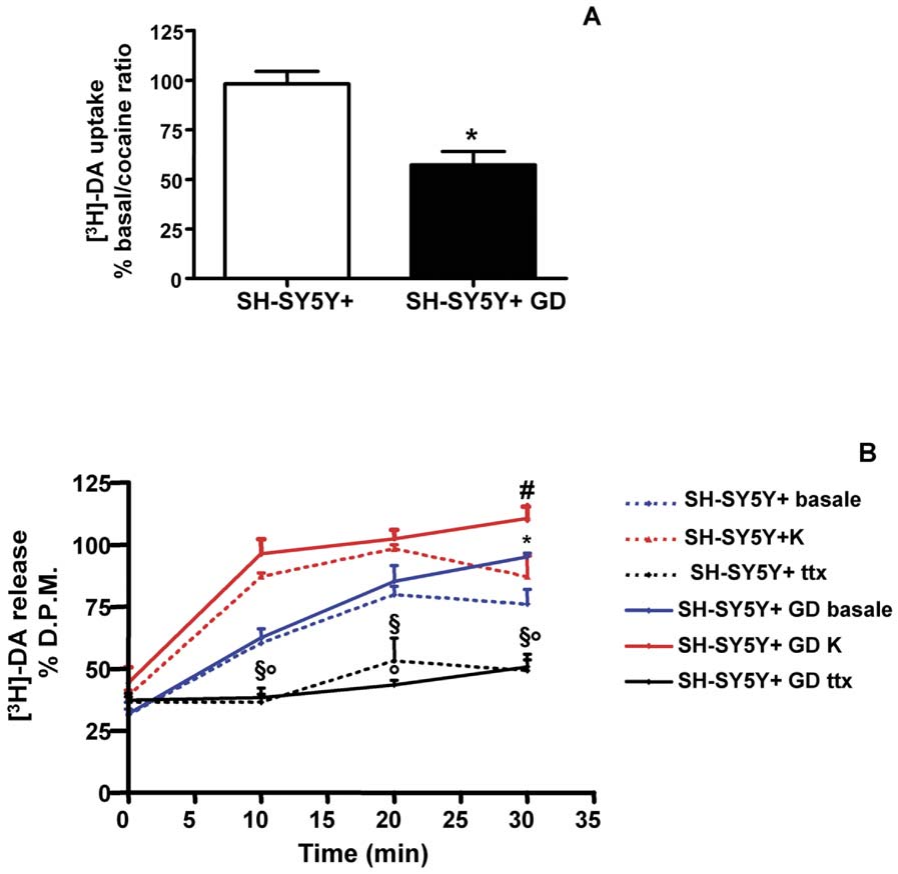
[^3^H]-DA uptake and [^3^H]-DA release in control and glucose-deprived SH-SY5Y+ cells. A: The graph is showing [^3^H]-DA uptake by SH-SY5Y+ and glucose deprived SH-SY5Y+ cells (SH-SY5Y+ GD). B: The graph is showing [^3^H]-DA release by SH-SY5Y+ and SH-SY5Y+ GD cells in basal contition and after TTX block or 50 mM K^+^ (K) treatment.

To evaluate whether the formation of DAT-α-synuclein complexes can coincide with a reduction of DA uptake we assayed % [^3^H]-DA uptake by SH-SY5Y+ and glucose deprived SH-SY5Y+ cells (fig. 7A). We found that in the SH-SY5Y+ subjected to GD the [^3^H]-DA uptake ratio was significantly lower when compared to untreated cells (* −42.77% P <0.05). The specificity of DA uptake by the DAT was assayed by using cocaine (please see figure S5A for further information).

Finally we evaluated % [^3^H]-DA release from SH-SY5Y+ and glucose deprived SH-SY5Y+ cells (Fig. 7B). [^3^H]-DA levels in the media, were normalized to the initial [^3^H]-DA quote which was taken by the cells (please see figure S5B for further information), and expressed as % changes. We found a significant reduction of extracellular [^3^H]-DA levels after 30 minutes in control SH-SY5Y+ cells (* −19%, P <0.01, Bonferroni's post comparison test) when compared to glucose deprived SH-SY5Y+ cells. The difference of [^3^H]-DA levels after 30 minutes was even higher between K^+^-treated SH-SY5Y+ and K^+^-treated-glucose deprived-SH-SY5Y cells (# −24%, P <0.01, Bonferroni's post comparison test). TTX treatment induced a significant block of [^3^H]-DA release in both SH-SY5Y+ (§ P <0.05 Bonferroni's post-comparison test) and glucose deprived SH-SY5Y+ cells (° P <0.05 Bonferroni's post-comparison test). Finally, we performed control experiments by evaluating basal, K^+^-stimulated, TTX-blocked as well as K^+^ and TTX treated SH-SY5Y and glucose deprived-SH-SY5Y cells in the presence of the DAT blocker cocaine (please see File S1 for further information). Remarkably, we found that glucose deprived SH-SY5Y+ cells showed reduced [^3^H]-DA release and that cocaine abolished the time dependent decrease of extracellular [^3^H]-DA levels in the media of SH-SY5Y+ cells (figure S5C).

## Discussion

Our study showed the occurrence of a redistribution of DAT/α-synuclein complexes visualized *in situ* by PLA in synaptic terminals as well in the cell soma of dopaminergic nigrostriatal neurons of the SYN120 transgenic mouse model. We also found a significant augment of striatal DAT levels, thus indicating that both DAT expression and distribution are crucially affected by the synaptic accumulation of the pathological C-terminally truncated (1–120) form of α-synuclein. Indeed, the DAT co-localized and interacted with α-synuclein into intracellular inclusions following the pathological aggregation of this latter in dopaminergic neurons of the nigrostriatal system of 12-month-old SYN120 transgenic mice. In agreement with previous observations showing that the residues 58–107 of α-synuclein are directly involved in the interaction with the C-terminus of the DAT [15], our data confirm that the carboxy-terminal truncation of α-synuclein doesn't alter the ability of the protein to bind the DAT.

Remarkably, our data showed a frank redistribution of DAT/α-synuclein PLA-positive complexes both in striatal terminals and in neuronal cell bodies of nigral dopaminergic neurons, indicating that α-synuclein may likely play a role, not only in the control of DAT trafficking in striatal synapses, but also in regulating DAT localization within the somatodendritic compartment of dopaminergic cells. These observations are in line with our previous findings showing that glucose deprivation, an insult which stimulates α-synuclein insoluble aggregation, induced the formation of DAT/α-synuclein-immunopositive inclusions within the cell bodies and processes of primary mouse mesencephalic dopaminergic neurons [14]. In the substantia nigra, the DAT can be specifically transported into dendrites. In particular, it is involved in the tuning of intracellular and extracellular DA levels in the somatodendritic compartment [49]. Therefore, our observations indicate that, whether α-synuclein aggregation may compromise DAT trafficking, this event may lead to alterations in somatodendritic DA release. As DA release by the somatodentritic compartment is a phenomenon that can usually facilitate motor functions in physiological conditions by mechanisms that may act independently from axon terminal DA release in the striatum, it may be feasible that one of the consequences of α-synuclein aggregation-dependent DAT redistribution within the dopaminergic neurons may be the impairment of this process. Hence, DAT redistribution may affect both striatal synaptic and somatodendritic DA release. This hypothesis is reinforced by the fact that we found that glucose deprived SH-SY5Y+ cells showed a reduction of [^3^H]-DA uptake and that this event was paralleled by a time-dependent accumulation of [^3^H]-DA in the medium of these same cells. Furthermore, we found that [^3^H]-DA release, evaluated in the presence of the DAT blocker cocaine increased in a time dependent manner in the SH-SY5Y+ cells. Conversely, in the glucose deprived cells cocaine did not induced a time-dependent increase in [^3^H]-DA release.

In line with results from a recent research report showing that density of DAT immunoreactivity in the dorsal striatum is significantly lower in the C57BL/6S mice when compared to C57BL/6J mice [18] we found that DAT levels were reduced in the striatum of C57BL/6S mice. Furthermore, our data showed that the overexpression of the truncated form of α-synuclein altered the striatal levels of the DAT. Indeed, we found a significant increase in DAT levels in the brain of the SYN120 mice when compared to the C57BL/6J and C57BL/6S mice, indicating that whether the lack of α-synuclein coincides with a reduction of DAT, the overexpression of its c-terminally truncated form induces a significant augment of DAT expression. However, it has to be considered that 12-month-old SYN120 mice show a significant reduction of both basal and depolarization-dependent striatal DA release as well as a decrease of DA metabolites [25] which is likely indicative of a reduction of DA turnover. Hence, we can't exclude that the increased expression of the DAT may be the consequence of compensatory mechanisms related to these alterations. Nonetheless, it could be feasible that this phenomenon may further exacerbate the formation of insoluble intracellular inclusions as the DAT is retained together with aggregated α-synuclein within synaptic terminals and neuronal cells. To date, it has been reported that DAT-positive neurons of the nigrostriatal system express low α-synuclein levels, suggesting that nigral dopaminergic neurons may be particularly vulnerable to variations of α-synuclein levels [50]. This is supported by other findings showing that siRNA knockdown of α-synuclein induced a significant 50% decrease of DAT activity in neuroblastoma cells [51] like the knock out of α-synuclein significantly decreases striatal DAT expression [18] and DA uptake. Thus, α-synuclein may act by promoting DAT expression in physiological conditions, although recent evidence has demonstrated that overexpression of α-synuclein is able to decrease the rate and magnitude of DAT-mediated substrate uptake [52]. Noteworthy, the data collected in the present study indicate that DAT expression and subcellular distribution may also be altered as a consequence of α-synuclein aggregation. Indeed, our findings suggest that the aggregation of α-synuclein could impair the correct trafficking of the DAT to synaptic sites. This hypothesis is supported by the fact that we recently demonstrated that α-synuclein and the DAT share common trafficking mechanisms and that as a consequence of the critical interaction between these proteins, agents which are able to modulate DAT trafficking to the plasma membrane, such as DA D2/D3 receptor agonists and cocaine, may also indirectly regualte α-synuclein localization [14]; [17]. On this line, it has been shown that disruption of the interaction of α-synuclein with microtubules enhances the cell surface recruitment of the DAT [17]. Remarkably, besides binding to tubulin [53], the main constituent of the microtubular network, α-synuclein can also interact with- and modulate the dynamics of actin cytoskeleton in physiological conditions [54]; [55], thus rendering it possible that its aggregation may consequently alter various synaptic processes. Hence, α-synuclein aggregation may result in a decrease of α-synuclein actin-binding properties, a phenomenon which may then alter actin cytoskeleton dynamics thus affecting DAT trafficking to synaptic sites. In this scenario, it could be feasible that a loss of the correct assembly of the cytoskeleton may lead to the critic accumulation of other synaptic vesicle associated proteins. In agreement with this hypothesis, we previously described the occurrence of a substantial redistribution of SNAREs in striatal synapses of SYN120 transgenic mice. It has to be taken into account that although the DAT has not been directly demonstrated to be present in vesicles, its membrane content is inhibited by toxins which are able to inhibit SNAREs function, while it is enhanced by SNAREs overexpression [23]; [56]–[58], thus the possibility that DAT redistribution could be also a consequence of SNAREs redistribution can't be excluded.

Finally, another important clue to take into account is that Afonso-Oramas and coworkers [59] recently showed that the DAT can be either redistributed from the plasma membrane to the endoplasmic-reticulum-Golgi compartment or persistently down regulated in response to slight or substantial dopaminergic lesions, respectively. Remarkably, from our previous findings we know that dopaminergic neurons of the nigrostriatal system in the 12 month-old SYN120 transgenic mice are hypofunctioning but do not degenerate as we couldn't find activation of the apoptotic or autophagic pathways or a decrease in TH levels [24]; [25]. This notwithstandings, we recently found that, in the brain 12 month-old SYN120 transgenic mice, the dopaminergic nigrostriatal neurons bearing α-synuclein inclusions show the activation of the unfolded protein response (UPR), an endoplasmic reticulum stress-related pathway which is induced by α-synuclein accumulation within the endoplasmic reticulum itself [26]. To date, it has been shown that prolonged activation of the UPR usually coincides with a block of endoplasmic reticulum-Golgi traffic and with the inhibition of the trans-Golgi network (TGN), a system which is crucially implicated in synaptic vesicles biogenesis and repackaging. In this scenario, whether the trans-Golgi network is impaired by the α-synuclein-induced UPR activation, the transfer of α-synuclein from the cell body to the synapse which is mediated by the fast axonal transport [43], vehiculating Golgi-derived vesicles containing neurotransmitters or associated proteins to the axolemma, may be critically reduced. This may lead to a stall of α-synuclein-related synaptic vesicle pools, with a substantial disassembling of the synaptic proteome. Therefore, we can't exclude that the redistribution of synaptic proteins observed in the striatum of these mice may be a consequence of these events. However, these aspects still need to be investigated.

### Conclusions

Taken together, our observations indicate that, in the early pathogenesis of PD α-synuclein accumulation may induce a redistribution of the DAT. Indeed, in line with the idea that α-synuclein is a causative agent for PD [60], we found that this protein plays a pivotal role in the regulation of this key protein involved in the function of dopaminergic synapses of the nigrostriatal system. These findings point out that α-synuclein accumulation can contribute to the onset of synaptic dysfunctions in dopaminergic neurons of the nigrostriatal system in the PD brain.

## Supporting Information

Figure S1 A: Representative photomicrograph showing α-synuclein expression (SYN-1 antibody) in SH-SY5Y+ cells, SH-SY5Y+ cells subjected to GD and SYN120-transfected as well as pcDNA1-transfected SH-SY5Y+ cells. B: The table is showing the quantitative analysis of the SYN-1-immunopositive bands in SH-SY5Y+ cells, SH-SY5Y+ cells subjected to GD and SYN120-transfected as well as pcDNA1-transfected SH-SY5Y+ cells. Please note the statistically significant increase (+1.4, P<0.01) of the SYN120/SYN140 ratio in the SYN120-transfected SH-SY5Y+ cells.
(TIF)

Figure S2 **Western blotting and immunoprecipitation studies on SH-SY5Y+ cells. 60 µg of proteins were loaded in the input and 150 µg of proteins were used for the immunoprecipitation experiments.** A: Representative immunoblotting showing DAT immunoprecipitation from plasma membrane extract of control, glucose deprived (SH-SY5Y+ GD) and SYN120-transfected (SH-SY5Y+ SYN 120). Amyloid precursor protein (APP) inputs from the respective membrane extracts were used as a control. B: Representative immunoblotting showing that DAT and synapsin 1 co-immunoprecipitated with α-synuclein in SH-SY5Y+ cells. C: Blots are showing that although CREB-2 was induced in SYN120-transfected glucose deprived-SH-SY5Y+ cells, it didn't co-immunoprecipitate with α-synuclein.
(TIF)

Figure S3 **Double immunofluorescent staining for DAT (panels B, F, J) and APP (A, E, I) in the substantia nigra of C57BL/6J, SYN120 and C57BL7/6S mice.** Please note that in the substantia nigra of the C57BL/6J and C57BL/6S mice DAT labelling showed a distribution that was similar to that of APP, while in the SYN120 transgenic mice it was mainly located in intracellular inclusions. Scale bar: A = 40 µm for A-L.
(TIF)

Figure S4 **Western blotting and immunoprecipitation studies on 12 month old SYN120, C57BL/6J and C57BL/6S mice.** 30 µg of proteins were loaded in the input and 100 µg of proteins were used for the immunoprecipitation experiments. A: Representative immunoblotting showing that the DAT co-immunoprecipitated with truncated α-synuclein in the striatum of 12 month old SYN120 and C57BL/6J mice. C57BL/6S mice were used as negative controls for co-immunoprecipitation.
(TIF)

Figure S5 A: [^3^H]DA uptake in SH-SY5Y+ and glucose deprived-SH-SY5Y+ cells in basal conditions and after cocaine treatment. Please note that the glucose deprived cells showed a statistically significantly decreased [^3^H]DA uptake (* −72 %, P <0.01, Bonferroni's post-comparison test) when compared to control SH-SY5Y+ cells. Cocaine treatment significantly blocked [^3^H]DA uptake in SH-SY5Y+ (# −105 %, P <0.001, Bonferroni's post-comparison test) and SH-SY5Y+ cells subjected to GD (§ −36 %, P <.01, Bonferroni's post-comparison test). B: % [^3^H]DA levels in the SH-SY5Y+ cell media, cell lysates and total values (indicative of the sum of [^3^H]DA levels in media and lysates) in SH-SY5Y+ and glucose deprived SH-SY5Y cells in basal conditions and after K^+^ and TTX treatment. C: [^3^H]DA release from SH-SY5Y+ and glucose deprived-SH-SY5Y+ cells in basal conditions and after K+ and/or TTX treatments. Please note that basal [^3^H]DA release from SH-SY5Y+ cells was higher than that observed in the glucose-deprived cells. Furthermore, [^3^H]DA release in the presence of cocaine was unable to induce a time-dependent increase in [^3^H]DA release in the glucose deprived cells.
(TIF)

File S1 **Supplementary information concerning the methods used for immunoprecipitation studies and for assaying [^3^H]DA release in the presence of cocaine.**
(DOC)
