# Correction: Redistribution of DAT/α-Synuclein Complexes Visualized by “In Situ” Proximity Ligation Assay in Transgenic Mice Modelling Early Parkinson's Disease

**DOI:** 10.1371/annotation/58679d17-c619-4f13-b11f-146dacfe7e92

**Published:** 2012-01-04

**Authors:** Arianna Bellucci, Laura Navarria, Elisa Falarti, Michela Zaltieri, Federica Bono, Ginetta Collo, Maria Grazia Spillantini, Cristina Missale, PierFranco Spano

The name of the seventh author in the article byline and the author contributions contained an error. The correct name is: Maria Grazia Spillantini. In the author contributions, the correction should be to the last contribution: "Wrote the paper: AB MGS CM PS." 

