# Supplementary figures and images for: Redistribution of DAT/α-Synuclein Complexes Visualized by “In Situ” Proximity Ligation Assay in Transgenic Mice Modelling Early Parkinson's Disease
Source: PLoS One. 2011 Dec 7;6(12):e27959. doi: 10.1371/journal.pone.0027959 (PMC3233557; doi:10.1371/journal.pone.0027959)

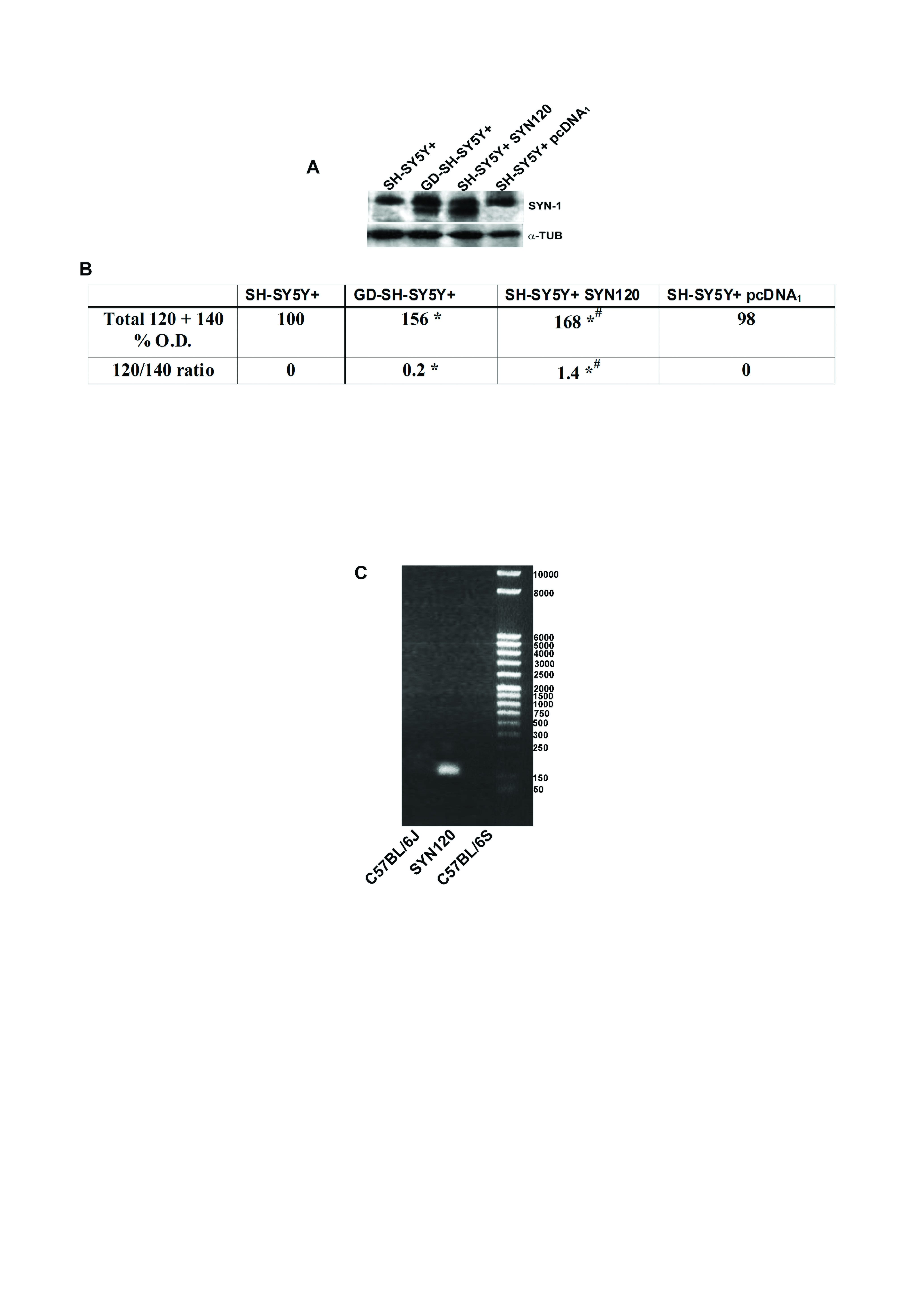

Supplement: Figure S1 — A: Representative photomicrograph showing α-synuclein expression (SYN-1 antibody) in SH-SY5Y+ cells, SH-SY5Y+ cells subjected to GD and SYN120-transfected as well as pcDNA1-transfected SH-SY5Y+ cells. B: The table is showing the quantitative analysis of the SYN-1-immunopositive bands in SH-SY5Y+ cells, SH-SY5Y+ cells subjected to GD and SYN120-transfected as well as pcDNA1-transfected SH-SY5Y+ cells. Please note the statistically significant increase (+1.4, P<0.01) of the SYN120/SYN140 ratio in the SYN120-transfected SH-SY5Y+ cells. (TIF) [file pone.0027959.s001.tif]

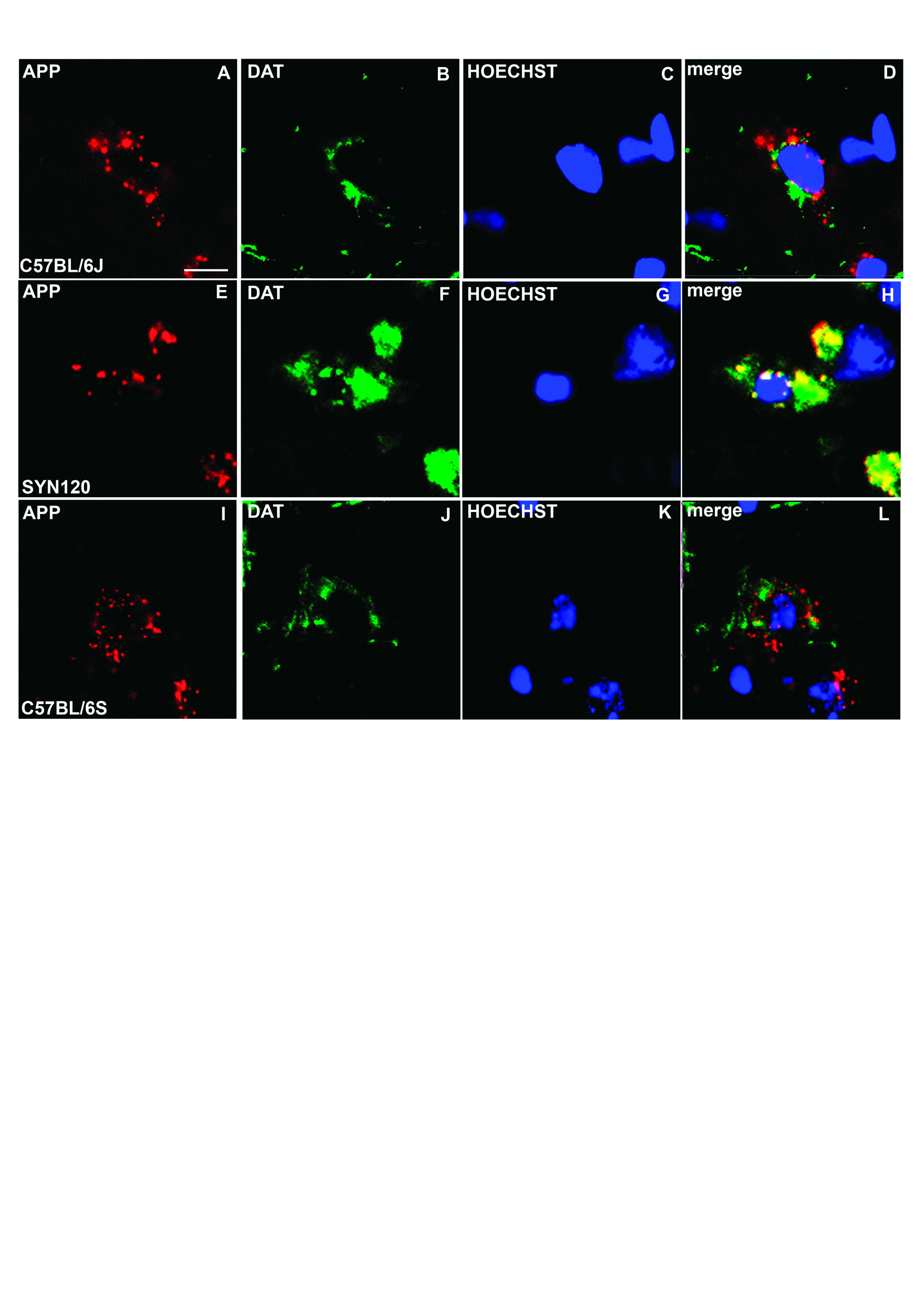

Supplement: Figure S3 — Double immunofluorescent staining for DAT (panels B, F, J) and APP (A, E, I) in the substantia nigra of C57BL/6J, SYN120 and C57BL7/6S mice. Please note that in the substantia nigra of the C57BL/6J and C57BL/6S mice DAT labelling showed a distribution that was similar to that of APP, while in the SYN120 transgenic mice it was mainly located in intracellular inclusions. Scale bar: A = 40 µm for A-L. (TIF) [file pone.0027959.s003.tif]

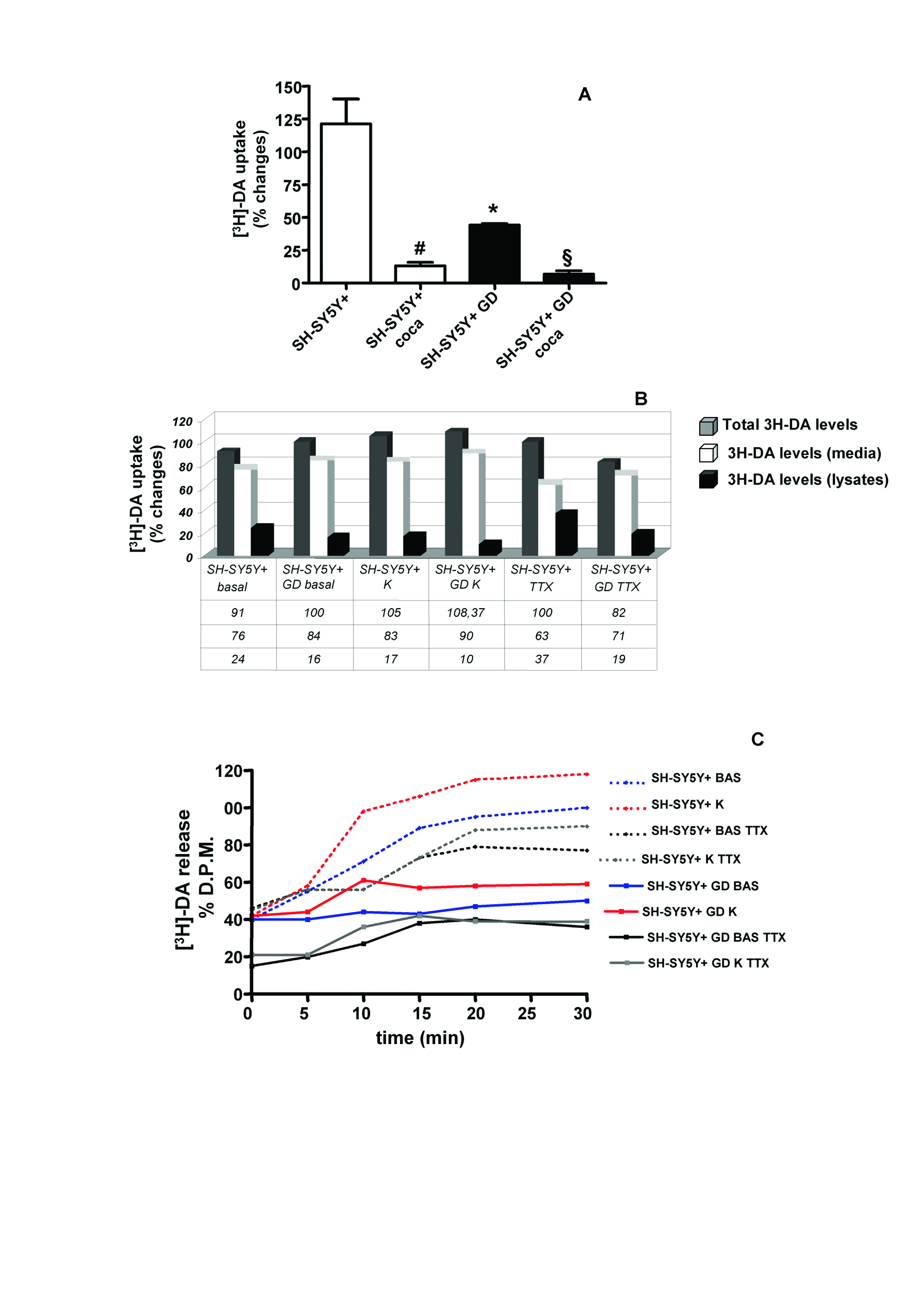

Supplement: Figure S5 — A: [3H]DA uptake in SH-SY5Y+ and glucose deprived-SH-SY5Y+ cells in basal conditions and after cocaine treatment. Please note that the glucose deprived cells showed a statistically significantly decreased [3H]DA uptake (* −72 %, P <0.01, Bonferroni's post-comparison test) when compared to control SH-SY5Y+ cells. Cocaine treatment significantly blocked [3H]DA uptake in SH-SY5Y+ (# −105 %, P <0.001, Bonferroni's post-comparison test) and SH-SY5Y+ cells subjected to GD (§ −36 %, P <.01, Bonferroni's post-comparison test). B: % [3H]DA levels in the SH-SY5Y+ cell media, cell lysates and total values (indicative of the sum of [3H]DA levels in media and lysates) in SH-SY5Y+ and glucose deprived SH-SY5Y cells in basal conditions and after K+ and TTX treatment. C: [3H]DA release from SH-SY5Y+ and glucose deprived-SH-SY5Y+ cells in basal conditions and after K+ and/or TTX treatments. Please note that basal [3H]DA release from SH-SY5Y+ cells was higher than that observed in the glucose-deprived cells. Furthermore, [3H]DA release in the presence of cocaine was unable to induce a time-dependent increase in [3H]DA release in the glucose deprived cells. (TIF) [file pone.0027959.s005.tif]
