# Supplementary material for: Redistribution of DAT/α-Synuclein Complexes Visualized by “In Situ” Proximity Ligation Assay in Transgenic Mice Modelling Early Parkinson's Disease
Source: PLoS One. 2011 Dec 7;6(12):e27959. doi: 10.1371/journal.pone.0027959 (PMC3233557; doi:10.1371/journal.pone.0027959)
Supplement: File S1 — Supplementary information concerning the methods used for immunoprecipitation studies and for assaying [3H]DA release in the presence of cocaine. (DOC) [file pone.0027959.s006.doc]

**Supplementary material**

**Immunoprecipitation protocol**

For co-immunoprecipitation (co-IP) studies equal amounts of total protein extracts (200 μg) were subjected to 1 h pre-clearing in ice-cold RIA buffer (NaCl 400 mM, EDTA 20 mM, Na_2_HPO_4_ 20 mM, 1% NP-40) with 20 µl A/G Plus Agarose beads (Santa Cruz Biotechnology). Samples were then centrifuged for 5 min at 1000 X g and the resulting supernatant was then incubated 2h under agitation at 4°C with the primary antibody at optimized dilution. Then, 30 µl Protein A/G Plus Agarose beads were added and the sample was incubated over night at 4°C. On the following day the samples were centrifuged at 1000 X g for 5 min at 4°C. Pellets were washed three times with RIPA buffer (Tris-HCl 50 mM, pH 7.4; 150 mM NaCl, 1% NP40, 0.25% Na deoxycholate, 0,1 mM PMSF) and then homogenized in 30 μl of sample buffer. For gel analysis, samples were boiled for 3 minutes, run on a 10% SDS-PAGE gel, blotted and probed with specific antibodies.

**[^3^H]DA release with cocaine block**

[^3^H]DA release was assessed in SH-SY5Y+ cells and SH-SY5Y+ cells subjected to GD. The medium was removed and cells were washed three times with 37°C Basal-Krebs-Ringer-Solution (B-KRS) (119mM NaCl, 2,5mM KCl, 2,5mM CaCl_2_, 1,3mM MgSO_4_, 1mM NaH_2_PO_4_, 26,2mM NaHCO_3_, 10mM glucose, 1mM L-ascorbic Acid, pH 7,4). The cells were pre-loaded with [^3^H]DA incubating 15 minutes at 37°C. Later, cells were quickly washed twice with B-KRS at 37°C and immediately treated in presence of 10µM cocaine respectively with B-KRS and K^+^-Krebs-Ringer-Solution (K^+^-KRS) (69mM NaCl, 50mM KCl, 2,5mM CaCl_2_, 1,3mM MgSO_4_, 1mM NaH_2_PO_4_, 26,2mM NaHCO_3_, 10mM glucose, 1mM L-Ascorbic Acid, pH 7,4) in presence or absence of 10µM tetrodotoxin (TTX). Every 5 minutes samples were collected for a total session of 30 minutes. At the end of 30 minutes was picked up whole medium and after two washes cells were collected in 1N NaOH. [^3^H]DA of all samples was quantified using scintillation cocktail in β-counter. The percentage of [^3^H]DA released in the medium was determined dividing the radioactivity accumulated in the medium by total [^3^H]DA uptake in the cells (released [^3^H]DA and cell fraction at the end of experiment).
